# Choice of generic antihypertensive drugs for the primary prevention of cardiovascular disease - A cost-effectiveness analysis

**DOI:** 10.1186/1471-2261-12-26

**Published:** 2012-04-04

**Authors:** Torbjørn Wisløff, Randi M Selmer, Sigrun Halvorsen, Atle Fretheim, Ole F Norheim, Ivar Sønbø Kristiansen

**Affiliations:** 1 Institute of Health and Society, University of Oslo, Oslo, Norway; 2 Norwegian Knowledge Centre for the Health Services, Oslo, Norway; 3 Norwegian Institute of Public Health, Oslo, Norway; 4 Department of Cardiology, Oslo University Hospital, Oslo, Norway; 5 University of Bergen, Bergen, Norway

## Abstract

**Background:**

Hypertension is one of the leading causes of cardiovascular disease (CVD). A range of antihypertensive drugs exists, and their prices vary widely mainly due to patent rights. The objective of this study was to explore the cost-effectiveness of different generic antihypertensive drugs as first, second and third choice for primary prevention of cardiovascular disease.

**Methods:**

We used the Norwegian Cardiovascular Disease model (NorCaD) to simulate the cardiovascular life of patients from hypertension without symptoms until they were all dead or 100 years old. The risk of CVD events and costs were based on recent Norwegian sources.

**Results:**

In single-drug treatment, all antihypertensives are cost-effective compared to no drug treatment. In the base-case analysis, the first, second and third choice of antihypertensive were calcium channel blocker, thiazide and angiotensin-converting enzyme inhibitor. However the sensitivity and scenario analyses indicated considerable uncertainty in that angiotensin receptor blockers as well as, angiotensin-converting enzyme inhibitors, beta blockers and thiazides could be the most cost-effective antihypertensive drugs.

**Conclusions:**

Generic antihypertensives are cost-effective in a wide range of risk groups. There is considerable uncertainty, however, regarding which drug is the most cost-effective.

## Background

Hypertension is a major risk factor for cardiovascular disease (CVD) such as acute myocardial infarction (AMI), stroke, heart failure and death. WHO has estimated that hypertension alone accounts for 4.4% of all disability adjusted life years that are lost [1]. An array of randomized controlled trials (RCTs) has demonstrated that antihypertensive drugs can reduce the risk of CVD. This is the case for thiazides, beta blockers, calcium channel blockers (CCB), angiotensin receptor blockers (ARB) and angiotensin-converting-enzyme inhibitors (ACE) [2]. Still, there is uncertainty and even controversy related to the intervention thresholds and the choice of first-line drug and "add-on" drugs. The controversy is partly related to the price of the different drugs, and partly to disagreements about how the available evidence on effectiveness, and side-effects of the various drugs should be interpreted [3,4].

The prices of the different antihypertensives vary, and price alone is only one factor which should be taken into account when considering which drugs that should be reimbursed. Several countries, including Norway, have chosen to use economic evaluation (cost-effectiveness analysis) for reimbursement decisions and development of guidelines. This implies that health authorities issue guidelines for choice of drugs and may even deny reimbursement of drugs that are too expensive in relation to the effectiveness. For example, the National Institute of Health and Clinical Excellence (NICE) in the UK may recommend against reimbursement of drugs when the cost per quality adjusted life year (QALY) exceeds £30,000 [5]. The argument for such thresholds is simply that if the costs of gaining a life year are beyond £30,000, resources may generate more health if they were spent elsewhere in the health care system. Cost-effectiveness analyses have been widely used for some types of therapies such as cholesterol lowering drugs. For antihypertensive therapies, however, relatively few studies have been published, especially during the last five years [6]. It is therefore a paucity of updated studies of the cost-effectiveness of such therapies.

A recent project funded by Norwegian health authorities offered a basis for developing guidelines for choice of antihypertensive drugs. The project first involved a comprehensive literature review and subsequent meta-analyses [2], and secondly, the development of a simulation model (Norwegian Cardiovascular Disease model (NorCaD)) [7] for economic evaluation. The aim of this study was to explore the life-time cost-effectiveness of various generic antihypertensive drugs in order to propose first-line therapy of hypertension and later add-ons for patients who need more than one drug. The scope of the project was restricted to primary prevention of CVD events. We assumed that patients first are offered dietary and other life style advice in order to achieve an acceptable blood pressure and that drugs are only prescribed when treatment goals are not reached with non-pharmacologic measures. We chose to use life years gained as the measure of health benefit because relatively few clinical trials report quality of life endpoints. We adopted a health care perspective which means that the analyses capture all costs that are incurred to the health care system.

## Methods

### Decision-analytic model

**Figure 1:**
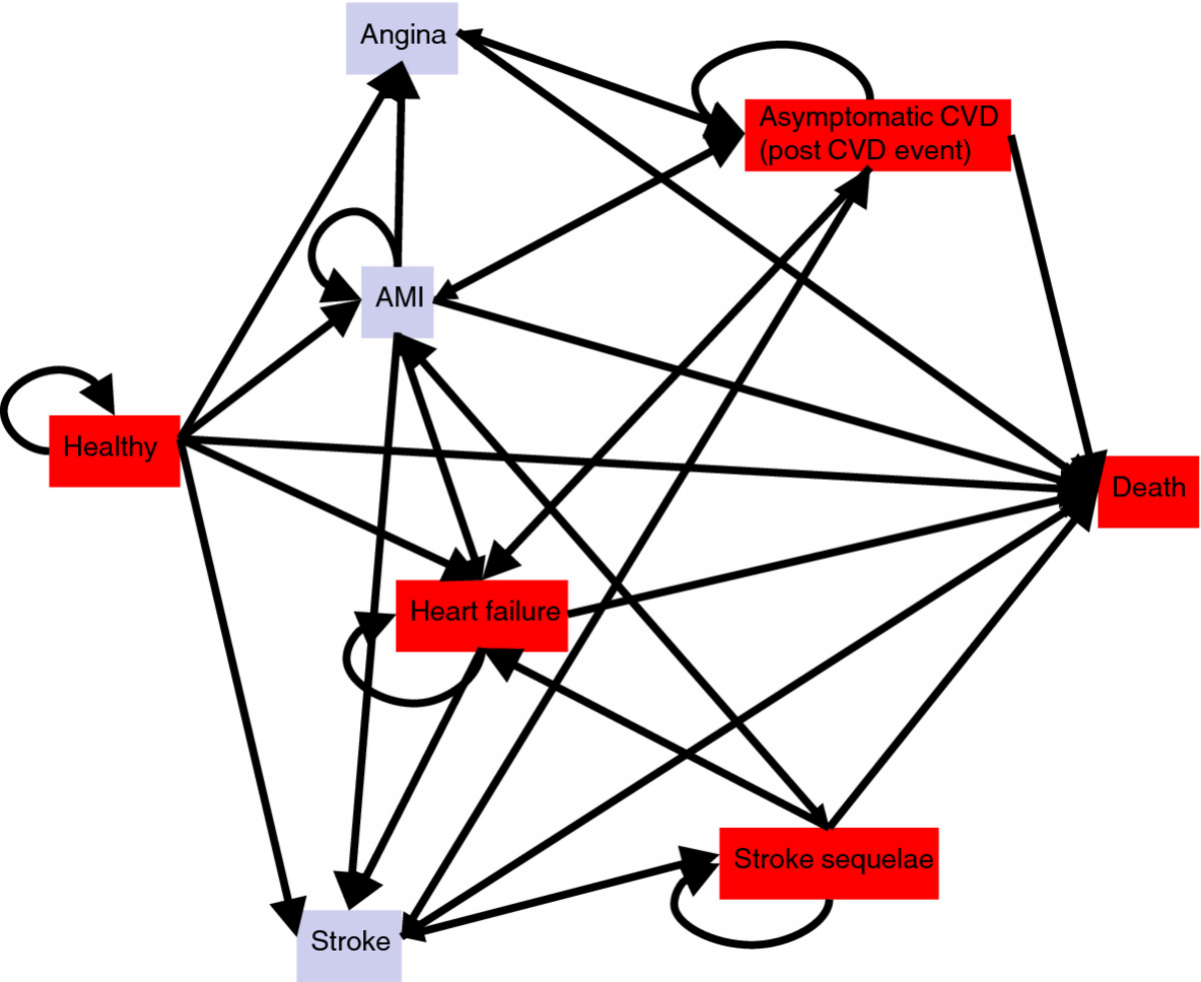
**Model structure**.

We used TreeAge Pro^® ^to develop a decision-analytic cardiovascular model which follows patients without prior cardiovascular incidents from the asymptomatic stage through their cardiovascular life to death [7]. Because CVD involves various types of disease events and health states, we chose to build a Markov model that follows individuals with different baseline characteristics (blood pressure, cholesterol level, *etc*.) until they all are dead or become 100 years (Figure 1). A full description of the model is available [7].

In the model, individuals start as "healthy" or "disease-free", *i.e*. without any prior cardiovascular event or symptoms. Disease-free individuals are subject to various primary CVD events: Acute myocardial infarction (AMI), stroke, heart failure, angina, death from cardiovascular disease. In addition, all individuals are at risk of death from other causes. The risks of these events were based on data from Norwegian registries and a Norwegian cohort study [8-10]. That is, we assumed that individuals follow the age- and sex specific incidence rates for the Norwegian population. After the first CVD event, patients either move directly into a CVD health state, or they may experience a secondary CVD event (*e.g*. reinfarction) and then move into a CVD health state. The NorCaD model consists of nine different health states: disease-free, heart failure, moderate stroke sequelae, severe stroke sequelae, post myocardial infarction, post angina, post stroke without sequelae, dead from cardiovascular causes and dead from other causes. Being alive in these health states, patients are at risk of worsening or of having secondary cardiovascular events (Figure 1). Figure 1 is a simplification as post MI, post angina and post stroke without sequelae are represented with one state "Asymptomatic CVD" (post CVD event). Moderate and severe sequelae are represented by stroke sequelae.

Patients who experience a secondary event are assumed to be in the health state which is worst. Hence if a patient with stroke sequelae experiences a myocardial infarction, costs and risks of an AMI is added, but thereafter the patient again has the risks and costs associated with the stroke sequelae and not those related to having asymptomatic CVD.

### Probabilities of events

The model was calibrated to predict Norwegian CVD specific mortality rates (for further details see technical report [7]) and we used life years gained as the measure of benefit in the analysis. In line with Norwegian guidelines for socioeconomic analyses, we used a 4% discount rate for both health outcomes and costs. In practice this implies that costs and health outcomes is valued 4% lower for each year. This approach of discounting is common within economic analyses to numerically describe the fact that most people rather would have money or good health today than in their future.

Most data on the probability of secondary events were based on international registries (EuroHeart [11-15] & GRACE [16,17], see also [7]), but RCT data were used when registry data were unavailable (see Tables 1 and 2). The risk of secondary events were dependent on time since primary event and age [7].

**Table 1:** Risk of secondary events during the first year after a primary event

Primary event	Secondary event	Probability of secondary event				Comment
			
		Value	Low	High	Time	
Angina	Cardiovascular death (men)	0.0108	0.0060	0.0156	One year	Daly et.al. (EuroHeart) [11]

Angina	Cardiovascular death (women)	0.0134	0.0071	0.0197	One year	Based on Daly et.al. (EuroHeart) [11]

Angina	AMI (men)	0.0153	0.0096	0.0211	One year	Based on Daly et.al. (EuroHeart) [11]

Angina	AMI (women)	0.0173	0.0101	0.0245	One year	Based on Daly et.al. (EuroHeart) [11]

Angina	Stroke (men)	0.0119	0.0069	0.0170	One year	Based on Daly et.al. (EuroHeart) [11]

Angina	Stroke (women)	0.0110	0.0053	0.0168	One year	Based on Daly et.al. (EuroHeart) [11]

Angina	Heart failure (men)	0.0153	0.0096	0.0211	One year	Based on Daly et.al. (EuroHeart) [11]

Angina	Heart failure (women)	0.0181	0.0108	0.0254	One year	Based on Daly et.al. (EuroHeart) [11]

AMI	Death (30-59 years)	0.04	0.074	0.106	One year	Swedish official data [18]

AMI	Death (60-69 years)	0.09	0.074	0.106	One year	Swedish official data [18]

AMI	Death (70-79 years)	0.20	0.074	0.106	One year	Swedish official data [18]

AMI	Death (80 years or more)	0.38	0.074	0.106	One year	Swedish official data [18]

Non-Stemi	Angina*	0.090	0.074	0.106	One year	ICTUS [19]

Non-Stemi	Heart failure	0.246	0.235	0.256	In-hospital	Fox, GRACE [16]

Non-Stemi	Reinfarction	0.014	0.011	0.017	In-hospital**	Hasdai, EuroHeart 1 [12]

Non-Stemi	Stroke	0.018	0.015	0.020	6 months***	Budaj, GRACE [17]

Non-Stemi	Stroke	0.009	0.007	0.011	In-hospital***	Budaj, GRACE [17]

STEMI	Angina*	0.114	0.083	0.145	One year	Zijlstra 1999 [20]

STEMI	Heart failure	0.288	0.277	0.298	In-hospital	Fox, GRACE [16]

STEMI	Reinfarction	0.027	0.022	0.032	In-hospital**	Hasdai, EuroHeart 1 [12]

STEMI	Stroke	0.021	0.018	0.023	6 months***	Budaj, GRACE [17]

STEMI	Stroke	0.013	0.011	0.015	In-hospital***	Budaj, GRACE [17]

Reinfarction	Stroke	Assumed to be the same as after STEMI/non-STEMI				

Reinfarction	Angina*	Assumed to be the same as after STEMI/non-STEMI				

Reinfarction	Death*	0.242	0.135	0.349	30 days	Andersen, DANAMI-2 [21]

Reinfarction	Heart failure	Assumed to be the same as after STEMI/non-STEMI				

Secondary heart failure	A heart failure llasts for 6-12 months	0.500	0.333	0.750		Expert opinion (SH)

Secondary heart failure	Death	0.290	0.240	0.340	One year	Based on EuroHeart 2 [13]

Primary heart failure	Death (men)	0.173	0.132	0.213	One year	Based on EuroHeart 2 [14]

Primary heart failure	Death (women)	0.163	0.116	0.209	One year	Based on EuroHeart 2 [14]

Stroke	Death	0.338	0.315	0.361	One year	Based on registry data Terent et.al. [22] and Kammersgaard et.al. [23]

Stroke	Moderate sequelae	0.072	0.060	0.084	One year	Based on registry data, Riks-Stroke [24]

Stroke	Severe sequelae	0.169	0.158	0.180	One year	Based on registry data, Riks-Stroke [24]

*Adjusted because data stems from RCTs

** In-hospital probabilities are assumed to be half of one-year-probabilities (1/3 - 1 in sensitivity analyses)

***Here exist both in-hospital and 6 month data (see formula under 2.2)

**Table 2:** Risk of new CVD events more than one year after first CVD event relative to healthy subjects

Health state	Secondary event	Probability of later event			Comment
			
		Value	Low	High	
Post AMI	AMI*	3.05	1.47	4,60	DANAMI-2 [25]

Post AMI	Angina*	21.7	15.8	27.6	Zijlstra [20]

Post AMI	Dying (30-59 years)*	3.55			OPTIMAAL [26], DANAMI-2 [25], and RIKS-HIA [18]

Post AMI	Dying (60-69 years)*	2.36			OPTIMAAL [26], DANAMI-2 [25], and RIKS-HIA [18]

Post AMI	Dying (70 years or more)*	1.00			OPTIMAAL [26], DANAMI-2 [25], and RIKS-HIA [18]

Post AMI	Stroke*	2.77	2.08	3.47	Zijlstra [20]

Post angina	AMI (men)	3.88	2.24	5.60	OPTIMAAL [26]

Post angina	AMI (women)	1.17	0.76	1.59	OPTIMAAL [26]

Post angina	Angina*	11.32	8.30	14.29	Assumed to be half of the probability first year, SMM-report nr 5/2002 [27]

Post angina	Death	1.23	0.82	1.65	Assumed to be half of the probability first year, SMM-report nr 5/2002 [27]

Post angina	Stroke (men)	5,34			NOKC-report nr 8/2004 [28]

Post angina	Stroke (women)	5,26			Based on meta-analyses from Nordmann [29] + HKS [9]

Post stroke	AMI	3.51	1.78	5.33	Risks based on relationship between angina and well first year after angina

Post stroke	Death	4.91	3.86	5.97	Risks based on relationship between angina and well first year after angina

Post stroke	Stroke	2.82	1.81	3.48	van Wijk [30]

Heart failure	dying 2nd year after HF (women)	6.67	6.16	11.04	van Wijk [30] and SSB [31]

Heart failure	dying 3rd year after HF (women)	7.61	5.08	10.15	van Wijk [30]

Heart failure	dying later years after HF (women)	2.45	0.90	4.00	Rosolova, Euroheart 2 [14]

Heart failure	dying 2nd year after HF (men)	5.05	3.24	6.86	Rosolova, Euroheart 2 [14]

Heart failure	dying 3rd year after HF (men)	4.62	2.90	6.33	Rosolova, Euroheart 2 [14] and SSB [31]

Heart failure	dying later years after HF (men)	2.13	0.96	3.31	Rosolova, Euroheart 2 [14]

Heart failure	Stroke*	6.80	3.40	13.61	Rosolova, Euroheart 2 [14]

Heart failure	Worsening of HF	9.58	9.04	10.13	Rosolova, Euroheart 2 [14] and SSB [31]

Heart failure	AMI after HF (men)	1.5	0.6	3.8	Based on SAVE [32] and SOLVD [33]

Heart failure	AMI after HF (women)	4.1	1.8	9.3	Cleland, Euroheart [15]

Moderate stroke sequelae	AMI	4.41	3.32	5.28	Based on Mosterd et.al. [34]

Moderate stroke sequelae	heart failure	2	1	4	Based on Mosterd et.al. [34]

Moderate stroke sequelae	New stroke	4.30	3.92	4.62	Based on meta-regression from Touzé et.al. [35]

Moderate stroke sequelae	Dying	2	1.5	2.5	Expert opinion (ISK)

Severe stroke sequelae	Dying	3	2.25	3.75	Meta-analysis of Hillen [36] and Caro [37]

*Adjusted down in the model because the data stems from RCT's

### Effectiveness

The effectiveness of antihypertensive drugs was modelled by reducing the risk of primary CVD events based on meta-analyses from a recent systematic review [2]. This systematic review followed common standards for systematic reviews according to the Norwegian Knowledge Centre for the Health Services, which is the centre responsible for Cochrane reviews and health technology assessments in Norway. The reason for choosing this review was that this is a recent review which is thoroughly performed by a qualified group of experts in both cardiology, evidence based medicine and pharmaceuticals. All searches for literature were performed by experienced librarians and the reviewing process was performed according to international standards. The systematic review included 33 trials with in total 210,394 patients comparing antihypertensive drugs against each other or against placebo.

For all analyses, we followed patients until all were 100 years old or dead.

We did not include any impact of antihypertensives on the risk of heart failure and angina as primary events in the base case analyses because these outcomes are "softer" and more difficult to measure uniformly in trials. We did, however, conduct scenario analyses with these two outcomes included. This strategy is in accordance with the reporting of the meta-analysis [2] upon which this study was based. There, angina and heart failure were classified as "secondary outcomes".

The ALLHAT trial weighted heavily in the meta-analysis of ACE inhibitors. This trial had a considerable proportion of African American patients, and they may respond less to ACE treatment than Caucasians [38]. The ACE meta-analysis was therefore conducted both with and without the African American patients (Table 3) [38]. In our base case analyses, we used the meta-analyses where African Americans were excluded from the ALLHAT study, while these were included in our scenario analyses. For all other meta-analyses which our efficacy data were based on, we refer to the mentioned HTA report [2].

**Table 3:** Relative risks of outcomes according to type of treatment (ACE inhibitor *vs *CCB and diuretics* *vs *ACE inhibitor) and outcome (based on meta-analyses)

ACE inhibitor vs. CCB (all included)			
**Outcome**	RCTs	Patients	RR (random), 95% CI
Mortality (total)	2	22,503	1.05 [0.98 til 1.11]

AMI	2	22,503	0.97 [0.84 til 1.13]
Stroke	2	22,503	1.13 [0.97 til 1.32]

Heart failure	2	22,503	0.85 [0.78 til 0.94]
Angina	1	18,102	1.07 [0.99 til 1.17]
**Diuretics vs. ACE inhibitor (all included)**			

Mortality (total)	3	31,249	1.00 [0.95 til 1.07]
AMI	2	30,392	1.01 [0.88 til 1.17]

Stroke	3	31,249	0.88 [0.80 til 0.98]

Heart failure	2	30,392	0.94 [0.71 til 1.24]
Angina	1	24,309	0.91 [0.85 til 0.98]

**ACE inhibitor vs. CCB (african american excluded)**			
Mortality (total)	2	16,080	1.03 [0.96 til 1.11]
AMI	2	16,080	0.96 [0.85 til 1.08]

Stroke	2	16,080	1.04 [0.92 til 1.19]
Heart failure	2	16,080	0.86 [0.80 til 0.93]

Angina	1	11,679	1.05 [0.95 til 1.15]
**Diuretics vs. ACE inhibitor (african american excluded)**			
Mortality (total)	3	22,670	1.04 [0.96 til 1.12]

AMI	2	21,813	1.02 [0.93 til 1.12]
Stroke	3	22,670	0.98 [0.86 til 1.12]

Heart failure	2	22,670	0.97 [0.90 til 1.05]
Angina	1	15,730	0.95 [0.87 til 1.04]

*In our analyses, we have used the meta-analysis results for diuretics for the effect of thiazides

When estimating the effectiveness of combinations of two or three drugs we assumed a multiplicative interaction between the drugs, as has been done in previous similar analyses [39,40]. If for example the relative risk reduction was 10% (relative risk 0.9) for one drug and 20% (relative risk 0.8) for the other, we assumed the combined effect represented 28% relative risk reduction (1-0.8*0.9 = 0.28).

### Costs

The costs were estimated for primary events, secondary events and health states separately. Data on the quantity of use of health care were estimated from published studies, treatment guidelines and expert opinions. Data on unit costs were based on price lists from the Norwegian Medicines Agency (drugs), Norwegian fee schedules (doctors and outpatient clinic visits) and DRG price lists from 2011 (in-hospital care) [41-44]. Unit costs and resource use were combined into cost parameters for each event and health state (Table 4). We chose the least expensive in each group of antihypertensive drugs with doses similar to those of the relevant trials. We measured all costs in 2011 Norwegian kroner (NOK) and expressed them in Euro (€1.00 = NOK8.01).

**Table 4:** Cost parameters (2011 Norwegian Kroner (NOK), €1.00 = NOK8.01)

Description	Value (€)
Cost of developing angina and have treatment	15,242

Cost of being in the state post MI for a year	331

Cost of being in the state post stroke for a year	292

Cost of being in the state post angina for a year	292

Cost of dying a cardiac death in hospital	5,169

Short term costs of developing heart failure	1,346

Cost of worsening of heart failure	4,598

Cost of one year with heart failure	3,569

Cost of living in the health state moderate stroke sequelae	6,436

Costs of treating a non-ST-elevated myocardial infarction	22,674

Cost of being in the health state severe stroke sequelae (*i.e*. one year in nursing home)	99,875

Cost of reinfarction	3,713

Costs treating an ST-elevated MI	22,674

Costs of getting stroke	23,546

Cost of one unit DRG	4,615

Cost of GP visits when receiving statin treatment (first year)	185

Cost of GP visits when receiving statin treatment (later years)	94

Cost of GP visits when receiving thiazide treatment (first year)	195

Cost of GP visits when receiving thiazide treatment (later years)	97

Yearly cost of thiazide (hydrochlorothiazide 12.5 mg*)	20

Yearly cost of ACE inhibitor (enalapril 20 mg)	58

Yearly cost of calsium channel blocker (Amlodipin 5 mg*)	30

Yearly cost of ARB (losartan 100 mg**)	73

Yearly cost of beta blocker (atenolol 50 mg***)	35

*For hydrochlorothiazide and amlodipin we assumed half a pill each day

**For losartan we assumed half a pill the first 6 days

***For atenolol, we assumed two pills per day

We undertook analyses for different patient groups according to age, sex and CVD risk factors. First, we ran the model for each patient group without treatment. Subsequently, we ran it with various single, double and triple drug therapies. For each drug regimen we calculated the additional (incremental) lifetime cost and additional life expectancy.

### Sensitivity analyses

The NorCaD model is based on approximately 300 parameters. Parameters with uncertainty were modelled as distributions rather than point values. It should be noted that uncertainty in this context means "parameter uncertainty" or "second order uncertainty" and not variability or heterogeneity across patients [45]. All probabilities and adherence rates were incorporated as beta distributions because they are restricted to values between 0 and 1 [46]. Unit costs and quantifications of cost items were assumed to follow gamma distributions because such data typically are skewed with a long right tale. Relative risks were incorporated as log-normal distributions due to the properties of the logarithm of relative risks. Monte Carlo simulation of the model was run with 10,000 iterations.

### Framework for priority setting

The result of an economic evaluation typically concludes that one therapy, as compared to another, has an incremental cost (IC) and an incremental effect (IE)(*e.g*. life years gained). Traditionally, the final result of the analysis has been expressed as an incremental cost-effectiveness ratio (ICER):

ICER = IC/IE

In order to maximize health (*e.g*. life years) within a given budget, decision makers ought to prioritize treatments with the lowest ICER's until the budget is exploited. In practice, most health care programmes have not been subject to economic analysis, and hence, pragmatic thresholds for the ICER are chosen. We assumed that this threshold (T) is €62,000 (≈NOK 500,000; €1 = NOK8.01) per life year gained as suggested by the Norwegian Directorate of Health [47].

On some occasions, however, the simple rule based on ranking ICER's results in suboptimal priority setting. We therefore based the choice of antihypertensive therapies on the so called "incremental net health benefit" (INHB) approach. The INHB has the following definition:

INHB = IE-IC/T

If for example a treatment on average generates 1.3 life years per patient, have lifetime costs of €31,000 (including off-sets from avoided events) and T is €62,000, then INHB = 1.3-31,000/62,000 = 0.8. With this formula, cost-effective strategies will have positive INHB. If different strategies are compared to the same baseline, the strategy with the highest INHB is regarded as the most cost-effective. When choosing between treatments, health outcome will be maximized by choosing on the basis of INHB. If INHB is negative, the therapy is not cost-effective and resources should be spent elsewhere [45].

## Results

**Figure 2:**
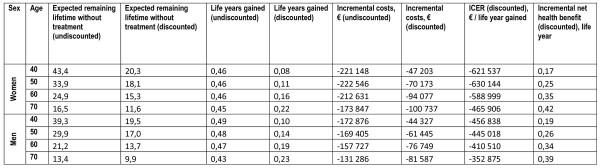
Incremental costs (€) and effects of CCB* compared to no treatment. *CCB = calcium channel blocker.

The model was run for four different age groups of women and men with average risk of CVD, that is following the national age- and sex-specific incidence rates from the registries. Firstly, CCB was compared to placebo (Figure 2), in which case treatment was dominant (less costly and higher effectiveness) in all age groups (40, 50, 60 and 70 years old). The (discounted) increase in life expectancy was similar for men and women, but higher for older age groups. The difference in (discounted) costs was also highest for the oldest age groups, which implies that INHB was highest for 70 year olds (0.42 and 0.39 for women and men respectively), and lower for the other age groups. All INHB's were positive based on a threshold for cost-effectiveness of €62,000 per life year gained.

To explore which drug is the most cost-effective first line antihypertensive treatment, we used head-to-head trials comparing drugs with CCB (Table 5). These analyses indicate that CCB may be the most cost-effective for all age groups (both men and women).

**Table 5:** Incremental net health benefit (life years) of different antihypertensive drugs compared with no treatment (Costs and life years discounted at 4%)

	Men				Women			
	**70**	**60**	**50**	**40**	**70**	**60**	**50**	**40**

ACE inhibitor	0,38	0,33	0,25	0,18	0,40	0,33	0,24	0,16

ARB	0,31	0,26	0,19	0,13	0,33	0,27	0,19	0,12

Beta blocker	0,22	0,19	0,14	0,10	0,23	0,19	0,14	0,10

CCB	**0,39**	**0,34**	**0,26**	**0,19**	**0,42**	**0,35**	**0,25**	**0,17**

Thiazide	0,37	0,32	0,25	0,18	0,39	0,33	0,24	0,16

*Comparisons were made through CCB, such that all analyses are based on head-to-head comparisons

**African Americans in the ALLHAT study excluded

Subsequently, we analyzed the choice of add-on treatment for patients who do not reach the treatment target. Here we compared CCB alone with any combination of two antihypertensive drugs. For all age groups, all combinations were dominant compared to CCB alone. The combination of CCB and thiazide yielded the greater positive incremental net health benefit, which indicates that this combination may be the most cost-effective combination of two drugs (Table 6). Hence, thiazide may be the most cost-effective first choice add-on treatment.

**Table 6:** Incremental net health benefit (life years) of various combinations of two antihypertensive drugs compared to CCB alone(Costs and life years discounted at 4%)

	Men				Women			
	**70**	**60**	**50**	**40**	**70**	**60**	**50**	**40**

ACE+beta	0,15	0,13	0,11	0,08	0,15	0,13	0,10	0,07

ACE+CCB	0,26	0,23	0,17	0,12	0,27	0,22	0,16	0,10

ACE+thia	0,25	0,22	0,17	0,12	0,25	0,21	0,15	0,10

ARB+ACE	0,20	0,17	0,12	0,08	0,21	0,16	0,11	0,07

ARB+beta	0,09	0,08	0,06	0,05	0,10	0,09	0,06	0,05

ARB+thia	0,20	0,17	0,13	0,09	0,21	0,17	0,12	0,08

CCB+ARB	0,20	0,17	0,13	0,09	0,22	0,17	0,12	0,08

CCB+beta	0,16	0,14	0,11	0,08	0,17	0,14	0,11	0,08

CCB+thia	**0,26**	**0,23**	**0,19**	**0,14**	**0,27**	**0,23**	**0,17**	**0,12**

Thia+bet	0,16	0,14	0,11	0,08	0,16	0,14	0,10	0,07

The subsequent analyses indicated that ACE inhibitor may be the most cost-effective second add-on drug when patients already use CCB and thiazide (Table 7). In this analysis, we also included other combinations of three drugs and none of these were more cost-effective than the combination of thiazide, CCB and ACE-inhibitors in any age group.

**Table 7:** Incremental net health benefit (life-years) of combinations of three antihypertensive drugs compared with CCB and thiazide (Costs and life years discounted at 4%)

	Men				Women			
	**70**	**60**	**50**	**40**	**70**	**60**	**50**	**40**

ARB+Bet+ACE	0,03	0,02	0,02	0,01	0,05	0,04	0,03	0,02

CCB+ARB+ACE	0,11	0,10	0,07	0,05	0,14	0,11	0,08	0,06

CCB+ARB+bet	0,03	0,02	0,01	0,01	0,05	0,04	0,03	0,02

CCB+Bet+ACE	0,08	0,07	0,05	0,04	0,10	0,08	0,06	0,04

Thia+ARB+ACE	0,11	0,10	0,08	0,05	0,13	0,11	0,08	0,05

Thia+ARB+bet	0,02	0,01	0,01	0,00	0,04	0,03	0,02	0,01

Thia+Bet+Ace	0,06	0,06	0,05	0,04	0,07	0,05	0,04	0,03

Thia+CCb+Ace	**0,15**	**0,13**	**0,11**	**0,08**	**0,17**	**0,14**	**0,10**	**0,07**

Thia+CCb+Bet	0,10	0,07	0,05	0,02	0,12	0,08	0,05	0,02

Thia+CCB+ARB	0,06	0,06	0,05	0,03	0,07	0,06	0,04	0,03

### Sensitivity and scenarios

**Figure 3:**
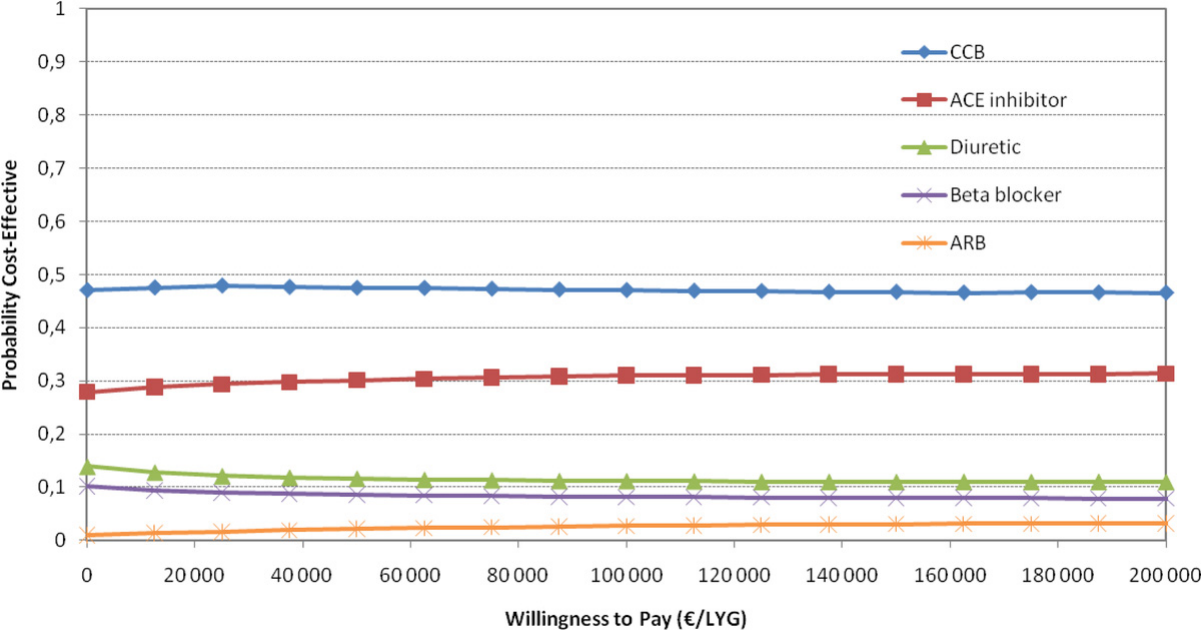
**Cost-effectiveness acceptability curves based on Monte Carlo simulations of single treatments and no treatment (data on effectiveness based on head-to-head-trials against CCB in addition to CCB vs placebo) -70 year old men (for simplicity; only curves with probability higher than 4% included in the graph)**.

**Figure 4:**
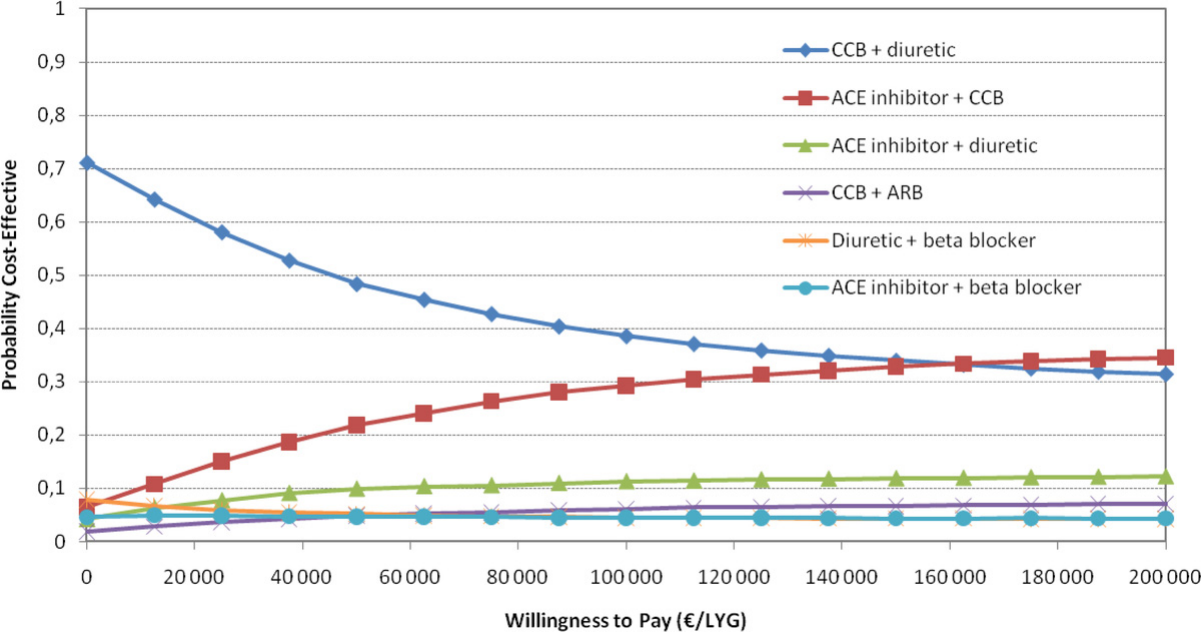
**Cost-effectiveness acceptability curves based on Monte Carlo simulations of combination treatments for 70 year old men (efficacy on heart failure and angina not included) (for simplicity; only curves with probability higher than 3% included in the graph)**.

**Figure 5:**
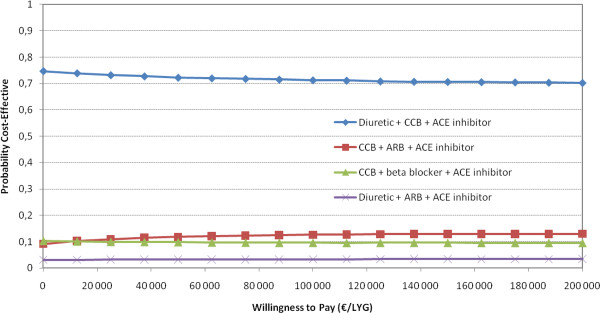
**Cost-effectiveness acceptability curves based on Monte Carlo simulations of combination treatments for 70 year old men (efficacy on heart failure and angina not included)**.

We performed probabilistic sensitivity analysis on the comparison of single drugs for 70 year old men. These analyses indicate substantial uncertainty with respect to which drug is the most cost-effective (Figure 3). No single drug was more than 50% likely to be the most cost-effective antihypertensive, regardless of what society is willing to pay for a life year gained. We also performed probabilistic sensitivity analysis on the choice of combination treatment of two or three drugs in 70 year old men (Figures 4 and 5). In Figure 5, there is a 72% probability that thiazide, CCB and ACE inhibitors are the most cost-effective combination of three drugs at a WTP of €62,000.

When African Americans from the ALLHAT study were included in meta-analyses, the conclusions were basically the same as when they were not (results not shown).

When we included heart failure and angina in the model, ARB was more likely than CCB to be the most cost-effective first-line treatment for hypertension (36% and 24% respectively)(data not shown). With angina and heart failure included, the choice of first add-on treatment was also more uncertain, even though a combination of either CCB, ARB or diuretics seemed most likely. In analyses of the combination of three drugs; all combinations seems almost equally likely of being the most cost-effective alternative if this more comprehensive model is used (results not shown).

## Discussion

For 40, 50, 60 and 70 year old men and women, generic CCB, thiazide, ACE inhibitor, ARB and beta blocker are all cost-effective and even cost-saving antihypertensives, even when two or all three are used in combination when this is indicated. CCB seems to be the most cost-effective alternative and consequently the first-line drug. If patients do not reach the treatment targets on CCB, thiazide is the most cost-effective add-on treatment. The sensitivity analyses, however, indicate considerable uncertainty in the ranking, and other factors such as side effects may well dictate the choice in the clinical setting.

The modelling showed lower incremental health benefit in younger than in older age groups. This may seem counterintuitive. To test this result, we performed validating analyses without discounting (Figure 2). From these, we concluded that the reason for the counterintuitive results is discounting. When the model was run without discounting, the incremental benefits are greater in younger age groups. Discounting decreases life years gained more in younger than in older age groups. Because benefits from antihypertensives in terms of life years gained occur at a late stage in life, discounting leads to lower incremental health benefit in the younger compared to the older age groups.

We used a 4% discount rate throughout this study, because this is currently recommended in Norwegian guidelines. We are aware, however, of the ongoing discussion regarding whether costs and/or effect should be discounted, and at what percentage [48,49]. If no discounting were applied, the age effects would be reversed in the sense that the ICERs would be more favourable for the young than the older age groups (Figure 2). If only costs were discounted, the difference in cost-effectiveness between age groups would be relatively small (Figure 2).

The estimated life year gains from taking CCB for the remainder of the lifetime compared to no antihypertensives is close to 0.5 (undiscounted). This may seem to be a small number when being 50 years old and expecting about 30 more years to live. Two points are worth mentioning, however. First, the 0.5 year is an average, and some patients may gain much more while others have no benefit. Some patients may die from other causes such as cancer or accidents and have no benefit from the antihypertensive treatment. Others may avoid a fatal stroke in their 60's and live until 80 because they started using antihypertensive drugs in their 50's. Second, 0.5 year is a considerable benefit in a public health perspective.

Models may be constructed in several different ways, and structural differences between them may in some cases result in considerably different results and alter the conclusions. Inclusion of heart failure and angina in the model made the results much more uncertain because the efficacy data are not consistent for these outcomes.

We have not identified other cost-effectiveness analyses of all these antihypertensives. However the UK guidelines from 2011 [50], which are based on similar efficacy documentation as our analyses, propose ACE inhibitor, CCB or possibly a low-cost ARB as the first choice. These guidelines also suggest that that a combination of CCB and either ARB or ACE inhibitor is the recommended combination of two drugs. If three drugs are to be combined, a thiazide-like diuretic is to be combined with the two-drug combination. These recommendations seem to fit well with our results.

Our analyses are based on meta-analyses from a recent systematic review [2]. The results are not very different from other meta-analyses [51,52]. Hence we assume that our results would not be substantially different if they were based on other meta-analyses of antihypertensive drug trials.

In our estimates of lifetime costs (e.g. Table 5), costs include consumption of all modelled resources. Hence, it is not possible to read drug prices directly from the undiscounted column of this table due to the complexity of NorCaD. This advantage with NorCaD helps avoid jumping to conclusions that the cheapest drug is the most cost-effective if no statistically significant evidence is available. For instance would a small decrease in rates of myocardial infarction more than outweigh a small increase in acquisition costs of a cheap generic drug.

### Strengths and limitations

The NorCaD model is comprehensive in the sense that it captures more CVD events and health states than most previous models. It is also a strength that the model is based on country-specific data for some of the crucial input parameters. The NorCaD model is designed primarily for primary prevention strategies for cardiovascular disease, and is therefore useful not only for statins and other pharmaceutical interventions, but also non-pharmaceutical interventions such as dietary advice and exercise.

While several previously published models have estimated the risk of CVD events on the basis of risk equations (typically the Framingham risk equations), we used observed incidence rates in the population and adjusted these rates up or down depending on the presence or absence of risk factors. The advantage of this approach is first that we avoid bias introduced by uncertainties in risk equations, and second that we avoid uncertainties introduced by distance in time or distance in geography. Our approach, however, is not without problems. Most important in this context is that we use register data for incidence rates, and there may be limitations in the quality of these registers and there may be inconsistencies between the registries.

The validation process proved that the input to the model need to be somewhat adjusted to fit Norwegian mortality data. This is a limitation of the model, which might be more consistent if it were based on fewer data sources, such as Framingham data. However, we considered the use of old data from the US likely to generate more bias.

Most of the trials that form our evidence base had duration of less than five years [2]. The life year gain generated through five years of treatment however is modest (usually less than 10% of the total gain). For the time beyond five years, we lack solid empirical data and simply assume that the relative effect stays constant. The assumption of "continued benefit" [53] is not necessarily true, and may overestimate the effect of treatment.

We have not incorporated side effects into our model, mainly because we use life years gained as measure of effectiveness and then possible fatal side effects will be captured in the clinical trials. The costs of side effects, however, may not be captured. Thiazide for example may have diabetogenic effect. Hence, thiazide may have a smaller effect and higher costs over time than what is assumed in our analyses. Some side effects may also have positive impact, such as the diuretic effect of thiazides. Whether incorporation of side effects would change the results of these analyses is uncertain, however. It should be noted, however, that side effects in terms of mortality is captured in the model because we used intention-to-treat data from trials. Such side effects are only omitted from the model if they occur after end of the trials.

As mentioned in the methods section, we assumed a multiplicative relationship when modelling combination treatment. Cohort studies have demonstrated an exponential relationship between blood pressure and CVD mortality. Thus one may argue that the risk reduction is proportional to the reduction in blood pressure. If one assumes that one drug results in a given reduction in blood pressure, independent of level, a combination of drugs will result in a multiplicative relationship. Even though some combination trials have been undertaken [2,54], these are too few to represent a basis for evaluating all clinical relevant combination therapies. Hence, the results with respect to combination therapy are more uncertain than those based with single drug comparisons.

The idea of a model is mimicking real life. All models are however to some extent a simplification of the clinical setting. In this model we chose not to include combinations of health states, such as for instance heart failure and stroke. In addition, we did not include all possible cardiovascular events, such as intermittent claudication. These simplifications would influence results to some extent, but we regarded the possible gain in accuracy to not be worth the hassle. This was mainly due to the fact that trustworthy data on occurrence and progression for these patients would be difficult to obtain.

## Conclusions

CCB, ARB, thiazide, ACE inhibitor and beta blocker all represent cost-effective antihypertensives either alone or in combination. Based on our findings, new clinical practice guidelines for antihypertensive treatment may do well in recommending more than one drug class as first choice.

## Competing interests

ISK has received honoraria from the Norwegian Ministry of Health, the Norwegian Directorate of Health, The Norwegian Medicines Agency, the Norwegian Diabetes Association, The Norwegian Knowledge Center for the Health Services, The South-East Regional Health Authority, and the pharmaceutical companies Glaxo, Merck, Nycomed and Pfizer. All these institutions may have an interest in antihypertensive policies. ISK does not own stocks in pharmaceutical companies, do not apply for patents and have not other conflict of interest than those stated above. The other authors (TW, RMS, SH, AF, OFN) declare that they have no competing interests.

## Authors' contributions

All authors contributed in writing the paper. TW, RMS, SH and ISK developed the model. All authors contributed to the choice of analyses and scope of the article. All authors have approved the final manuscript.

## Pre-publication history

The pre-publication history for this paper can be accessed here:

http://www.biomedcentral.com/1471-2261/12/26/prepub
